# Off-Hours Endoscopic Management and Clinical Outcomes in Acute Esophageal Variceal Bleeding: A Real-World Cohort Study

**DOI:** 10.3390/medicina62071272

**Published:** 2026-06-30

**Authors:** Fatih Kıvrakoğlu, Abdullah İlhan, Duran Deha Çetin, Mustafa Harı, İbrahim Erdem, Bünyamin Sarıtaş, Şehmus Ölmez

**Affiliations:** Department of Gastroenterology, Adana City Training and Research Hospital, Health Sciences University, 01230 Adana, Turkey; aggassp@gmail.com (A.İ.); dehacetin@gmail.com (D.D.Ç.); mustafa_hari@hotmail.com (M.H.); erdem_610@hotmail.com (İ.E.); bunyamine@hotmail.com (B.S.); drsehmusolmez@gmail.com (Ş.Ö.)

**Keywords:** weekend effect, esophageal varices, gastrointestinal hemorrhage, liver cirrhosis, endoscopy timing

## Abstract

*Background and Objectives*: Acute esophageal variceal bleeding is a life-threatening gastrointestinal emergency associated with high morbidity and mortality. Endoscopy plays a central role in its management; however, the impact of off-hours endoscopic care on clinical outcomes remains controversial. This study aimed to compare regular-hours and off-hours endoscopic management in patients with acute esophageal variceal bleeding. *Materials and Methods*: This retrospective single-center cohort study included adult patients who underwent endoscopy for acute esophageal variceal bleeding between January 2020 and January 2025. Patients were divided into regular-hours and off-hours groups according to the timing of endoscopy. Demographic characteristics, laboratory findings, liver disease severity scores, endoscopy timing, and clinical outcomes were retrospectively evaluated. The primary endpoint was in-hospital mortality. Secondary endpoints were early rebleeding, length of hospital stay, and 6-week mortality. *Results*: A total of 253 patients were included; 160 (63.2%) underwent endoscopy during regular hours, and 93 (36.8%) during off-hours. Ascites was more frequent in the off-hours group (73.1% versus 58.8%; *p* = 0.022), and albumin levels were lower (2.7 versus 3.1 g/dL; *p* = 0.024). The groups were similar in terms of age, sex, cirrhosis etiology, Child-Pugh-Turcotte class, and MELD-Na score. Most patients (90.3%) underwent endoscopy within 12 h, with no significant difference between groups. In-hospital mortality, 6-week mortality, early rebleeding, and length of hospital stay were also comparable. In multivariable logistic regression analysis adjusted for ascites, albumin level, and hepatic encephalopathy, off-hours endoscopic intervention was not independently associated with in-hospital mortality. *Conclusions*: Off-hours endoscopic management was not associated with worse mortality or rebleeding outcomes in acute esophageal variceal bleeding. These findings suggest that in well-organized centers with continuous endoscopy availability, clinical outcomes may be preserved regardless of the time of endoscopic intervention.

## 1. Introduction

Acute esophageal variceal bleeding is one of the most serious life-threatening complications of liver cirrhosis and requires urgent endoscopic treatment. Despite major advances in treatment, 6-week mortality rates remain approximately 10–20% [1]. Current international guidelines recommend a multidisciplinary approach, including hemodynamic stabilization, vasoactive therapy, prophylactic antibiotics, and urgent endoscopy within the first 12 h after hemodynamic stabilization [2,3,4,5].

The timely and high-quality implementation of this approach is largely influenced by hospital-related organizational factors, leading to the concepts of the “weekend effect” or “off-hours effect” [6]. This phenomenon suggests that patients admitted during weekends or outside regular working hours may experience worse outcomes due to reduced staffing, lower availability of experienced specialists, and delays in access to diagnostic or therapeutic procedures [6,7,8,9,10,11]. However, some studies have reported that the weekend effect does not lead to a significant difference in mortality [12,13,14,15].

The effect of systemic delays in access to resources during off-hours on major clinical outcomes such as mortality remains controversial. Although some meta-analyses have reported a slight increase in weekend mortality among patients with upper gastrointestinal bleeding, this effect has not been observed in acute esophageal variceal bleeding [16,17]. On the other hand, a recent study suggested that very early endoscopy, for example, within the first 6 h, may be associated with higher mortality compared with endoscopy performed within 6–24 h. This finding is generally explained by the fact that the most unstable patients are often taken to emergency endoscopy before adequate stabilization is achieved [18]. Similarly, some meta-analyses have found no significant differences in mortality or rebleeding between urgent (≤12 h) and delayed (>12 h) endoscopy in acute esophageal variceal bleeding [19].

Given the organizational and economic challenges of maintaining continuous 24 h endoscopy services, it is important to clarify whether off-hours endoscopic management truly worsens patient outcomes in acute esophageal variceal bleeding. However, the number of studies directly comparing regular-hours and off-hours endoscopic management in acute esophageal variceal bleeding remains limited.

In this retrospective study, we evaluated whether off-hours endoscopic management was associated with clinical outcomes among patients presenting to the emergency department with acute esophageal variceal bleeding in a high-volume referral center operating with a 24/7 active on-call system and experienced endoscopy teams. Our main hypothesis was that regular-hours and off-hours endoscopic management would yield comparable results in terms of major clinical outcomes, particularly in-hospital mortality and rebleeding.

## 2. Materials and Methods

### 2.1. Study Design and Patient Population

This study was conducted in a high-volume referral center with a dedicated endoscopy unit providing 24/7 emergency services, staffed by on-call gastroenterology fellows, gastroenterology specialists, and endoscopy nurses.

**Figure 1:**
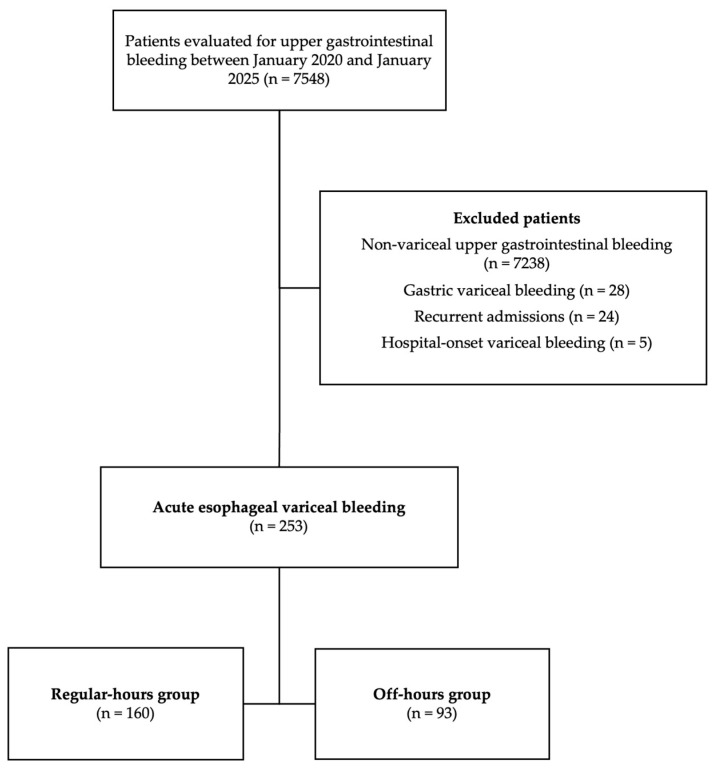
Flowchart of the patient selection process.

Patients aged 18 years or older with chronic liver disease who presented to the emergency department of Health Sciences University, Adana City Training and Research Hospital, with acute esophageal variceal bleeding between 1 January 2020 and 1 January 2025 were included. Patients with gastric variceal bleeding or non-variceal upper gastrointestinal bleeding, recurrent admissions, or missing clinical or laboratory data were excluded. Patients with gastric variceal bleeding were excluded because their hemodynamic characteristics, bleeding risks, and primary endoscopic management (typically cyanoacrylate glue injection rather than band ligation) differ significantly, which would introduce heterogeneity into our standardized treatment protocol. A flowchart of the patient selection process, including screened, excluded, and finally included patients, is presented in Figure 1.

Acute esophageal variceal bleeding was defined as presentation with signs of upper gastrointestinal bleeding, such as hematemesis, melena, or hematochezia, together with endoscopic evidence of active variceal bleeding, stigmata of recent bleeding, including nipple sign, adherent clot, red spot, cherry-red spot, or varix-on-varix appearance, or the presence of blood in the stomach with esophageal varices and no other identifiable bleeding source [2,3].

Early rebleeding was defined according to the APASL criteria [3]. Rebleeding within days 0–5 after initial hemostasis was considered present if at least one of the following occurred: new-onset hematemesis or melena, a decrease in hemoglobin level of ≥2 g/dL, requirement for additional blood transfusion, or signs of hemodynamic instability, defined as systolic blood pressure < 90 mmHg or heart rate > 100 beats/min [2,3].

All patients presenting with suspected acute esophageal variceal bleeding were managed according to a standard protocol based on the Baveno VII consensus guidelines [2]. Red blood cell transfusion was administered to maintain a target hemoglobin level of 7–8 g/dL. Vasoactive agents, such as terlipressin or somatostatin, were initiated immediately at admission and continued for 2–5 days. All patients received prophylactic broad-spectrum antibiotic therapy, ceftriaxone, at admission. After hemodynamic stabilization, upper gastrointestinal endoscopy (PENTAX Imagina EPK-i5500c; PENTAX Medical, Tokyo, Japan) was ideally performed within the first 12 h. The categorization of admission-to-endoscopy time into <12 h, 12–24 h, and >24 h was driven by current international guideline recommendations. Specifically, the Baveno VII consensus strongly advocates for urgent endoscopy within the first 12 h of presentation, making it the critical threshold for our primary comparative analysis [2,19]. Endoscopic variceal band ligation (CDBL01; Clinodevice, Istanbul, Turkey) was used as the primary endoscopic treatment. Transjugular intrahepatic portosystemic shunt (TIPS) was considered in patients who did not respond to endoscopic treatment or had persistent hemodynamic instability. All discharged patients were started on non-selective beta-blocker therapy, carvedilol or propranolol, for secondary prophylaxis. In addition, patients were enrolled in an endoscopic band ligation program with follow-up endoscopies scheduled at 4–6-week intervals until variceal eradication was achieved.

The sedation strategy during endoscopic procedures was determined according to the patient’s clinical status, procedural tolerance, and timing of presentation (regular hours or off-hours). Patients with preserved consciousness, no active hematemesis, and adequate procedural tolerance underwent endoscopy without anesthesiology support. Patients with impaired consciousness, poor procedural tolerance, or active hematemesis underwent endoscopy after airway protection with endotracheal intubation. This approach was performed in collaboration with an anesthesiologist during regular working hours and with an emergency physician during off-hours.

For patients with more than one hospitalization during the study period, only the first admission was included in the analysis to avoid statistical bias.

Data were retrospectively obtained from the electronic medical records of the Health Sciences University, Adana City Training and Research Hospital. The collected variables included age, sex, etiology of cirrhosis, and compensated or decompensated status of cirrhosis. Cirrhosis etiologies were classified as hepatitis B virus (HBV), hepatitis C virus (HCV), alcohol-related liver disease, metabolic dysfunction-associated steatotic liver disease (MASLD), cryptogenic cirrhosis, autoimmune liver disease, and other causes. To prevent misclassification, patients were categorized based on their primary dominant etiology. Secondary or overlapping etiologies (e.g., concurrent alcohol use in viral hepatitis) were not sub-classified as distinct categories to maintain clear stratification. The presence of ascites, hepatic encephalopathy, portal vein thrombosis, and acute kidney injury was also recorded.

Laboratory parameters evaluated at admission included a complete blood cell count (hemoglobin, white blood cell count [WBC], platelet count, neutrophil count, and lymphocyte count), coagulation profile (international normalized ratio [INR]), and biochemical parameters (urea, creatinine, alanine aminotransferase [ALT], aspartate aminotransferase [AST], total bilirubin, and albumin). In addition, Child-Turcotte-Pugh class (CTP), Model for End-Stage Liver Disease-sodium score (MELD-Na), AST-to-platelet ratio index (APRI), Fibrosis-4 index (FIB-4), and neutrophil-to-lymphocyte ratio (NLR) were calculated. All scores and classifications were assessed using data obtained at the time of initial presentation.

The timing of endoscopy was classified as regular hours or off-hours. The time interval between emergency department admission and endoscopy was also recorded. Clinical outcomes included in-hospital mortality, 6-week mortality, early rebleeding (0–5 days), and length of hospital stay.

Patients were divided into two groups according to the timing of endoscopy: regular-hours and off-hours groups. Regular-hours endoscopy was defined as procedures performed on weekdays between 08:00 and 17:00. Off-hours endoscopy was defined as procedures performed on weekdays between 17:00 and 08:00, as well as those performed on weekends and official holidays.

Early rebleeding was defined as the development of hematemesis or melena within 5 days after the initial bleeding episode, accompanied by a decrease in hemoglobin level. In-hospital mortality was defined as death occurring during hospitalization. Six-week mortality was defined as death occurring within 6 weeks after admission. Mortality data were obtained from the hospital electronic medical record system and the national death notification system.

The primary endpoint of the study was in-hospital mortality. Secondary endpoints were early rebleeding, length of hospital stay, and 6-week mortality.

Our hospital is a high-volume referral and training center. The gastroenterology endoscopy unit provides 24/7 service with experienced gastroenterology specialists, gastroenterology fellows, and endoscopy nurses. Gastroenterology fellows are on in-hospital duty, while specialists provide on-call coverage. All endoscopic procedures are performed either by gastroenterology specialists or by gastroenterology fellows under specialist supervision. All specialist physicians have at least 4 years of experience in therapeutic endoscopic procedures.

### 2.2. Statistical Analysis

Categorical variables were summarized as frequencies and percentages. The distribution pattern of continuous variables was evaluated before group comparisons; variables without normal distribution were reported as medians with minimum and maximum values. Comparisons between the regular-hours and off-hours groups were performed using Pearson’s chi-square test for categorical variables, whereas Fisher’s exact test was applied when cell counts were insufficient. Continuous variables were compared using the Mann–Whitney U test because the normality assumption was not fulfilled.

Variables showing significant differences in univariable analyses, together with clinically relevant covariates selected according to the previous literature, were entered into a multivariable logistic regression model. Before model construction, potential interaction effects and multicollinearity were assessed. Correlation matrices were reviewed, and variables with correlations exceeding 40% were examined to reduce multicollinearity. Model fit was assessed using the Hosmer–Lemeshow goodness-of-fit test and Nagelkerke R^2^. A two-tailed *p*-value below 0.05 was considered statistically significant. Statistical analyses were conducted using IBM SPSS Statistics for Windows, version 31.0 (IBM Corp., Armonk, NY, USA).

### 2.3. Ethical Approval

The study was approved by the Clinical Research Ethics Committee of Adana City Hospital (Decision No: 1330; Date: 30 April 2026). The study was conducted in accordance with the principles of the Declaration of Helsinki. Due to the retrospective design of the study, the requirement for informed consent was waived by the ethics committee.

## 3. Results

### 3.1. Patient Characteristics

A total of 253 patients were included in the study; 160 patients (63.2%) were in the regular-hours group, and 93 patients (36.8%) were in the off-hours group. In the overall cohort, the median age was 62 years (range: 22–88), and 34.4% of the patients were female. Regarding the etiology of cirrhosis, MASLD was the most common cause (31.6%), followed by HBV infection (26.1%), alcohol-related liver disease (16.6%), HCV infection (8.3%), cryptogenic cirrhosis (7.5%), and autoimmune hepatitis (5.5%).

Overall, 66% of the patients had a history of prior decompensating events (e.g., history of ascites or hepatic encephalopathy) before the index bleeding episode. Ascites was present in 64% of patients, hepatic encephalopathy in 15%, portal vein thrombosis in 11.9%, and acute kidney injury in 6.3%. Regarding liver disease severity, 24.9% of patients were classified as CTP class A, 58.1% as CTP class B, and 17% as CTP class C.

There were no significant differences between the regular-hours and off-hours groups in terms of age, sex, cirrhosis etiology, distribution of compensated/decompensated cirrhosis, hepatic encephalopathy, portal vein thrombosis, CTP classification, numerical CTP score, MELD-Na, APRI, FIB-4, or NLR (all *p* > 0.05). The median numerical CTP score was 7 (range, 5–14) in the regular-hours group and 8 (range, 5–13) in the off-hours group (*p* = 0.118). However, ascites was significantly more frequent in the off-hours group (*p* = 0.022). Acute kidney injury was significantly more common in the regular-hours group than in the off-hours group (*p* = 0.009). Demographic and baseline patient characteristics are shown in Table 1.

### 3.2. Laboratory Findings

When laboratory parameters were evaluated, no significant differences were observed between the groups in terms of urea, creatinine, total bilirubin, AST, ALT, white blood cell count, hemoglobin, platelet count, neutrophil count, lymphocyte count, or INR. In contrast, albumin levels were significantly lower in the off-hours group (*p* = 0.024). Laboratory findings are compared in Table 2.

### 3.3. Impact of Admission-to-Endoscopy Timing on Clinical Outcomes

When endoscopy timing was evaluated, most patients underwent endoscopy within the first 12 h (90.3%). Although the proportion of patients who underwent endoscopy within 12 h was higher in the off-hours group, this difference did not reach statistical significance (*p* = 0.230). In-hospital mortality and 6-week mortality rates were similar between the two groups (*p* = 0.495). In addition, no significant differences were observed in the length of hospital stay or early rebleeding rates (*p* = 0.933). It should be noted that the identical total numbers for in-hospital and 6-week mortality (*n* = 52) are coincidental. One patient who died during a prolonged hospitalization (on day 44) was included only in the in-hospital mortality group, while another patient who died post-discharge within the 42-day follow-up was included only in the 6-week mortality group. Consequently, the absolute counts remained the same despite a reciprocal difference in one patient between the cohorts. Endoscopy timing and clinical outcomes are compared in Table 3.

### 3.4. Multivariable Analysis of Factors Associated with In-Hospital Mortality

In the multivariable logistic regression analysis, numerical CTP score was independently associated with in-hospital mortality (OR: 1.85 per 1-point increase; 95% CI: 1.52–2.25; *p* < 0.001). In contrast, off-hours endoscopic intervention was not independently associated with in-hospital mortality (OR: 0.61, 95% CI: 0.29–1.28, *p* = 0.190). The multivariable logistic regression analysis is presented in Table 4.

## 4. Discussion

The “weekend effect” is a phenomenon described in various acute medical conditions, in which admission during off-hours is associated with worse outcomes [8,20,21]. In this context, it is particularly important to understand the factors that may influence outcomes in patients with acute esophageal variceal bleeding managed during off-hours. In our study, no significant differences were observed between the regular-hours and off-hours groups in terms of in-hospital mortality, 6-week mortality, early rebleeding, or length of hospital stay. Similarly, most patients underwent endoscopy within the first 12 h, and no significant difference was observed between the groups regarding time to endoscopy. Although the prevalence of ascites was higher and albumin levels were lower in the off-hours group, the groups were comparable in terms of CTP class and MELD-Na score. Moreover, after adjustment for numerical CTP score as a continuous measure of liver disease severity, off-hours endoscopic intervention was not independently associated with in-hospital mortality.

Myers et al. were the first to demonstrate that, despite a slight delay in endoscopy among patients admitted on weekends with acute esophageal variceal bleeding, mortality rates were similar to those observed in patients admitted on weekdays [13]. Similarly, Byun et al. reported no significant difference in 6-week survival, despite a longer waiting time for endoscopy during weekends [12]. The comparable clinical outcomes observed with regular-hours and off-hours management in acute esophageal variceal bleeding may be related to the highly standardized management of this condition. The rapid initiation of critical treatments, such as vasoactive therapy, antibiotic prophylaxis, and early hemodynamic stabilization, regardless of the time of day, may reduce the potential impact of delays in endoscopy [4,13,22].

Similar findings have also been reported in a more recent large-scale study. Sodoma et al. found no significant difference in in-hospital mortality between weekend and weekday admissions among patients presenting with acute esophageal variceal bleeding, although length of hospital stay was longer in the weekend group [23]. In contrast, a large-scale study by Merza et al. reported a slight increase in mortality among patients admitted on weekends [24].

When evaluating the off-hours effect, the optimal timing of endoscopy is also an important issue of debate. Although current guidelines, such as Baveno VII, recommend endoscopy within the first 12 h after hemodynamic stabilization, many clinicians still believe that earlier endoscopy may lead to better outcomes [2]. In the meta-analysis by Luo et al., endoscopy performed within the first 6 h was associated with higher mortality compared with endoscopy performed within 6–24 h [18]. Another meta-analysis, including nine retrospective studies and 2824 patients, also found that early endoscopy (<12 h) was associated with significantly higher mortality than delayed endoscopy (>12 h) [25]. This finding is generally explained by the fact that the most hemodynamically unstable patients may undergo emergency endoscopy before adequate stabilization is achieved [19]. In our study, 90.3% of patients underwent endoscopy within the first 12 h. No difference was observed between the regular-hours and off-hours groups in terms of time to endoscopy. This may be related to the presence of an experienced on-call endoscopy team at our center, which provides 24/7 active service for the management of acute esophageal variceal bleeding and is able to perform emergency endoscopic interventions without delay when necessary.

In the literature, prognostic scores such as CTP and MELD-Na are emphasized as among the strongest predictors of mortality in acute esophageal variceal bleeding [12,26,27]. In our analysis, the prevalence of ascites was significantly higher, and albumin levels were lower in the off-hours group. However, these differences were not reflected in prognostic scores, and no significant difference was observed between the groups in terms of CTP class or MELD-Na score. These findings suggest that, despite some baseline clinical differences in off-hours presentations, the overall severity of liver disease was similar between the two groups. In addition, multivariable logistic regression analysis showed that off-hours endoscopic intervention was not an independent predictor of mortality. In the recent literature, non-invasive fibrotic indices have been highlighted for their prognostic value; for instance, Tian et al. demonstrated the clinical utility of APRI and FIB-4 in predicting bleeding risk and 30-day prognosis in patients with esophagogastric varices. In our cohort, baseline APRI and FIB-4 values were comparable between the off-hours and regular-hours groups, further confirming that baseline fibrotic burden and disease severity were evenly matched regardless of admission time [28]. This suggests that not only baseline clinical characteristics and prognostic scores, but also appropriate follow-up and standardized treatment strategies, may have a determining and protective role in mortality among patients with acute esophageal variceal bleeding. The ability to perform emergency endoscopic interventions without delay and to maintain standardized treatment protocols through an experienced 24/7 endoscopy team in our center may have contributed to these results.

When the literature is reviewed, studies evaluating the role of off-hours care and endoscopy timing in early rebleeding after acute esophageal variceal bleeding show heterogeneous results. Most studies have reported that off-hours admission or endoscopy timing has no significant effect on early rebleeding rates, defined as rebleeding within 0–5 days [12,18,19,22]. However, some studies have shown a significant association between off-hours admission and early rebleeding [8]. In our study, no significant difference was found between the regular-hours and off-hours groups in terms of early rebleeding rates. This may be explained by the similar time to endoscopy between the groups, the fact that most patients underwent endoscopy within the first 12 h, the similarity of prognostic scores reflecting clinical disease severity, and the sustained implementation of standardized treatment protocols by an experienced team providing 24/7 service in our center.

In our study, the 6-week mortality rate was 20.6%. This rate was close to the upper limit of the 15–20% mortality range reported in the literature for acute esophageal variceal bleeding. This may be related to the fact that our study was conducted in a high-volume referral center and that the patient population largely consisted of patients with decompensated advanced-stage liver disease.

In our multivariable analysis, the most important factor determining mortality in patients presenting with variceal bleeding was not the timing of intervention, whether regular hours or off-hours, but the overall severity of liver dysfunction at presentation. In particular, the numerical Child-Turcotte-Pugh (CTP) score emerged as the most critical independent predictor (OR: 1.85 per 1-point increase, 95% CI: 1.52–2.25, *p* < 0.001), indicating that each 1-point increase in the CTP score is associated with an 85% increase in the odds of in-hospital mortality. This finding is strongly consistent with the existing literature showing that clinical outcomes in acute esophageal variceal bleeding are driven more by baseline liver reserve and comprehensive indices of organ failure, such as the CTP score, than by endoscopy timing [12,29,30].

Our study has several important strengths. A review of the literature shows that most studies evaluating the regular-hours and off-hours effect have limited their comparisons to weekdays and weekends. However, this approach generally excludes weekday night hours from the definition of off-hours. In our study, weekday night hours and official holidays were also included in the off-hours period. In this respect, our study provides a more comprehensive and realistic definition of the off-hours time period. In addition, to the best of our knowledge, this is one of the first studies to compare endoscopic management during regular hours and off-hours in patients with acute esophageal variceal bleeding, which increases the novelty of our study.

Although some of our patients underwent interventional procedures, particularly transjugular intrahepatic portosystemic shunt (TIPS), these data were not analyzed separately. It may be assumed that patients treated during off-hours could have more limited access to advanced interventional procedures such as TIPS. A possible reason for this is that interventional radiology services in our hospital operate through an on-call system rather than an active in-house duty system. Nevertheless, the absence of a significant difference in mortality between the groups suggests that access to advanced interventional treatments could be maintained during off-hours.

It is worth noting that the final cohort of acute esophageal variceal bleeding represents a relatively small fraction of the total screened upper gastrointestinal bleeding cases. This lower-than-expected proportion is primarily attributed to the strict exclusion criteria, which removed gastric varices, recurrent admissions, and hospital-onset bleeds, combined with the extremely high volume of non-variceal bleeding cases typical for our region’s largest emergency center.

Our study also has several limitations. First, this was a single-center study conducted in a single region; therefore, the findings may not be fully generalizable to different regions or healthcare system settings. Due to the retrospective design, not all clinical variables and potential confounding factors, particularly operator-dependent factors, could be fully controlled. The APASL 2026 guideline defines time zero (T0) in acute variceal bleeding as the onset of hematemesis [3]. In our study, T0 was accepted as the time of hospital admission; therefore, the effects of pre-hospital treatment-related factors on outcomes could not be evaluated. Another limitation of our retrospective design is the absence of detailed non-hepatic comorbidity profiles (e.g., specific cardiovascular diseases or concurrent malignancies) and dynamic bleeding severity parameters, such as the shock index. As emphasized by Marmo et al. in their evaluation of upper gastrointestinal bleeding severity, incorporating such hemodynamic and comorbidity indices alongside liver-specific scores could provide a more comprehensive assessment of patient risk and potential confounders [31]. Future prospective studies that record the actual onset time of bleeding and pre-hospital administration of vasoactive therapy may enable more accurate timing analyses. In addition, advanced rescue therapies such as TIPS were not analyzed separately. Since the study was conducted in a high-volume center with experienced staff, the generalizability of the findings to lower-volume centers or centers with limited resources may be restricted.

## 5. Conclusions

Our study demonstrated that there were no significant differences between regular-hours and off-hours endoscopic management in terms of mortality, rebleeding, or length of hospital stay in patients with acute esophageal variceal bleeding. Crucially, the absence of substantial differences in mortality and rebleeding between the regular-hours and off-hours cohorts is a testament to the effectiveness of a highly standardized management pathway. These results indicate that when continuous 24/7 endoscopy services are coupled with strict adherence to evidence-based protocols—such as immediate vasoactive therapy and prophylactic antibiotics—the quality of care and patient survival can be successfully maintained at a consistently high level during night shifts, weekends, and holidays.

## Figures and Tables

**Table 1:** Demographic and baseline patient characteristics.

Variable	Regular-Hours Group (*n* = 160)	Off-Hours Group (*n* = 93)	All Patients (*n* = 253)	*p* Value
**Age**	62 (26–85)	61 (22–88)	62 (22–88)	0.431 ^a^
**Female sex**	57 (35.6)	30 (32.3)	87 (34.4)	0.587 ^b^
**Etiology**				0.225 ^b^
HBV	43 (26.9)	23 (24.7)	66 (26.1)	
HCV	13 (8.1)	8 (8.6)	21 (8.3)	
Autoimmune hepatitis	8 (5.0)	6 (6.5)	14 (5.5)	
Alcohol-related liver disease	27 (16.9)	15 (16.1)	42 (16.6)	
MASLD	47 (29.4)	33 (35.5)	80 (31.6)	
Cryptogenic cirrhosis	17 (10.6)	2 (2.2)	19 (7.5)	
Other	5 (3.1)	6 (6.5)	11 (4.3)	
**Compensation** **status**				0.915 ^b^
Compensated	54 (33.8)	32 (34.4)	86 (34.0)	
Decompensated	106 (66.3)	61 (65.6)	167 (66.0)	
**Ascites**	94 (58.8)	68 (73.1)	162 (64.0)	**0.022 ^b^**
**Hepatic** **encephalopathy**	27 (16.9)	22 (23.7)	49 (19.4)	0.204 ^c^
**Portal vein** **thrombosis**	12 (7.5)	6 (6.5)	18 (7.1)	0.754 ^b^
**Acute kidney** **injury**	15 (9.4)	1 (1.1)	16 (6.3)	**0.009 ^b^**
**CTP**				0.412 ^b^
**Numerical CTP score, median** **(min–max)**	7 (5–14)	8 (5–13)		0.118
CTP-A	44 (27.5)	19 (20.4)	63 (24.9)	
CTP-B	91 (56.9)	56 (60.2)	147 (58.1)	
CTP-C	25 (15.6)	18 (19.4)	43 (17.0)	
**MELD-Na**	12 (7–39)	13 (6–37)	13 (6–39)	0.974 ^a^
**APRI**	0.9 (0.2–27.1)	0.8 (0.2–13.7)	0.8 (0.2–27.1)	0.744 ^a^
**FIB-4**	4.2 (0.5–43.5)	4.7 (0.5–37.2)	4.4 (0.5–43.5)	0.950 ^a^
**NLR**	4.9 (0.6–52.2)	5.1 (0.9–36.3)	5.0 (0.6–52.2)	0.912 ^a^

*Notes*: Data are shown as *n* (%) or median (minimum–maximum), as appropriate. ^a^ Mann–Whitney U test; ^b^ Pearson’s chi-square test; ^c^ Fisher’s exact test. *Abbreviations*: HBV: hepatitis B virus; HCV: hepatitis C virus; MASLD: metabolic dysfunction-associated steatotic liver disease; CTP: Child-Turcotte-Pugh; MELD-Na: Model for End-Stage Liver Disease-sodium; APRI: aspartate aminotransferase-to-platelet ratio index; FIB-4: Fibrosis-4 index; NLR: neutrophil-to-lymphocyte ratio.

**Table 2:** Laboratory findings of the patients.

Laboratory Parameter	Regular-Hours Group (*n* = 160)	Off-Hours Group (*n* = 93)	All Patients (*n* = 253)	*p* Value
Urea (mg/dL)	54.5 (6–308)	67 (10–271)	57 (6–308)	0.208 ^a^
Creatinine (mg/dL)	0.8 (0.3–7.0)	0.8 (0.3–3.8)	0.8 (0.3–7.0)	0.642 ^a^
Albumin (g/dL)	3.1 (1.4–5.9)	2.7 (1.5–4.4)	3.0 (1.4–5.9)	**0.024 ^a^**
Total bilirubin (mg/dL)	1.2 (0.2–28.7)	1.1 (0.1–17.7)	1.2 (0.1–28.7)	0.489 ^a^
ALT (U/L)	24 (5–269)	24 (6–542)	24 (5–542)	0.504 ^a^
AST (U/L)	39 (12–812)	43 (13–843)	40 (12–843)	0.538 ^a^
WBC (10^3^/µL)	8.2 (2.3–60.5)	8.7 (1.6–25.5)	8.4 (1.6–60.5)	0.644 ^a^
Hemoglobin (g/dL)	8.6 (3.9–14.8)	8.2 (3.6–14.1)	8.5 (3.6–14.8)	0.378 ^a^
Platelet count (10^3^/µL)	125 (26–424)	120 (23–589)	122 (23–589)	0.954 ^a^
Neutrophil count (10^3^/µL)	5.8 (1.1–33.0)	6.6 (1.2–22.0)	5.9 (1.1–33.0)	0.515 ^a^
Lymphocyte count (10^3^/µL)	1.2 (0.1–6.2)	1.2 (0.2–5.3)	1.2 (0.1–6.2)	0.944 ^a^
INR	1.4 (1.0–3.6)	1.4 (0.9–6.3)	1.4 (0.9–6.3)	0.877 ^a^

*Note*: Data are shown as *n* (%) or median (minimum–maximum), as appropriate. ^a^ Mann–Whitney U test. *Abbreviations*: ALT: alanine aminotransferase; AST: aspartate aminotransferase; WBC: white blood cell count; INR: international normalized ratio.

**Table 3:** Endoscopy timing and clinical outcomes.

Variable	Regular-Hours Group (*n* = 160)	Off-Hours Group (*n* = 93)	All Patients (*n* = 253)	*p* Value
**Admission-to-endoscopy time**				0.230 ^c^
<12 h	142 (88.8)	88 (94.6)	230 (90.3)	
12–24 h	14 (8.8)	3 (3.2)	17 (6.7)	
>24 h	4 (2.5)	2 (2.2)	6 (2.4)	
**In-hospital mortality**	35 (21.9)	17 (18.3)	52 (20.6)	0.495 ^b^
**6-week mortality**	35 (21.9)	17 (18.3)	52 (20.6)	0.495 ^b^
**Length of hospital stay**	5 (0–23)	5 (1–44)	5 (0–44)	0.763 ^a^
**Early rebleeding**	29 (18.7)	17 (18.3)	46 (18.5)	0.933 ^b^

*Notes*: Data are shown as *n* (%) or median (minimum–maximum), as appropriate. ^a^ Mann–Whitney U test; ^b^ Pearson’s chi-square test; ^c^ Fisher’s exact test.

**Table 4:** Multivariable logistic regression analysis of risk factors associated with in-hospital mortality.

Variable	OR	95% CI	*p* Value
**Off-hours endoscopic intervention**	0.61	0.29–1.28	0.190
**CTP score, per 1-point increase**	1.85	1.52–2.25	<0.001

Model fit: Hosmer–Lemeshow test *p* = 0.078; Nagelkerke R^2^ = 0.196. Abbreviations: CTP, Child–Turcotte–Pugh; OR, odds ratio; CI, confidence interval.

## Data Availability

The anonymized data underlying the findings of this study may be obtained from the corresponding author upon a justified academic request. Public deposition of the dataset was not possible because the data contain patient-level clinical information and are subject to institutional privacy restrictions.

## Abbreviations

ALT	Alanine aminotransferase
APASL	Asian Pacific Association for the Study of the Liver
APRI	Aspartate aminotransferase-to-platelet ratio index
AST	Aspartate aminotransferase
CI	Confidence interval
CTP	Child-Turcotte-Pugh
FIB-4	Fibrosis-4 index
HBV	Hepatitis B virus
HCV	Hepatitis C virus
INR	International normalized ratio
MASLD	Metabolically-dysfunction-associated steatotic liver disease
MELD-Na	Model for End-Stage Liver Disease-sodium
NLR	Neutrophil-to-lymphocyte ratio
OR	Odds ratio
T0	Time zero
TIPS	Transjugular intrahepatic portosystemic shunt
WBC	White blood cell count
